# Longitudinal hematologic decline during peginterferon alfa-2b therapy in chronic hepatitis B: clinical safety and exploratory whole-blood transcriptomics

**DOI:** 10.3389/fmed.2026.1919392

**Published:** 2026-10-26

**Authors:** Jie Li, Weilai Chen, Jinggu Chen, Jiayi Xing, Qi Chen, Jianhua Zhang, Ruixin Shi, Chenwei Pan, Yongping Chen

**Affiliations:** 1 Department of Infectious Diseases and Hepatology, The Second Affiliated Hospital and Yuying Children's Hospital of Wenzhou Medical University, Wenzhou, Zhejiang, China; 2 Zhejiang Provincial Key Laboratory for Accurate Diagnosis and Treatment of Chronic Liver Diseases, the First Affiliated Hospital of Wenzhou Medical University, Wenzhou, Zhejiang, China; 3 Wenzhou Key Laboratory of Precision General Practice and Health Management, Wenzhou, Zhejiang, China

**Keywords:** chronic hepatitis B, hematologic safety, leukopenia, neutropenia, peginterferon alfa-2b, thrombocytopenia, transcriptomics, whole-blood RNA-seq

## Abstract

Peginterferon-based therapy for chronic hepatitis B is known to cause leukopenia, neutropenia, and thrombocytopenia, but the relationship of early hematologic trajectories to serological response, supportive management, and whole-blood molecular signals remains uncertain. We conducted an exploratory hematologic safety-focused secondary analysis of a prospective, single-center cohort of 21 patients receiving peginterferon alfa-2b-based therapy (13 responders and 8 non-responders). WBC, ANC, and platelet counts were assessed at visits corresponding approximately to baseline, Week 5, and Week 10. Non-parametric comparisons were supplemented with effect sizes and 95% confidence intervals, and supportive-treatment timing was examined in locked sensitivity analyses. All paired Visit-1-to-Visit-3 changes were negative in both response groups, whereas responder-versus-non-responder estimates were imprecise and did not establish a response-group-specific toxicity pattern. Visit-2 WBC <4.0 × 10^9^/L occurred in 19/21 patients (90.5%; exact 95% CI: 69.6%-98.8%) and was treated as a pragmatic clinical-management context threshold rather than a discriminating transcriptomic endpoint. A post-documented oral-support-date indicator did not materially alter the fitted WBC or ANC pattern, but this non-randomized sensitivity analysis cannot estimate treatment efficacy. In the manuscript-consistent DESeq2 variance-stabilizing-transformation workflow, no unadjusted primary Hallmark or candidate-gene family met the prespecified exploratory BH-FDR *q* < 0.10 threshold. A reviewer-directed xCell2 sensitivity analysis showed longitudinal internal concordance between the prespecified neutrophil enrichment score and measured ANC; composition adjustment changed several pathway estimates, but no paired W0-W10 family reached *q* < 0.10 and unstable baseline-adjusted platelet signals did not support a specific pathway or mechanism. These small-cohort findings are exploratory, are not evidence of equivalence or mechanism, and require confirmation in larger independent cohorts.

## Introduction

Peginterferon-based therapy remains one of the few finite treatment options for selected patients with chronic hepatitis B, particularly in treatment strategies that emphasize durable immune control and functional-cure-oriented endpoints (1–3). At the same time, interferon-based therapy has a clinically relevant adverse-effect profile that requires close monitoring and active management. Leukopenia, neutropenia, and thrombocytopenia are among the best-recognized safety concerns and can influence treatment tolerability, monitoring intensity, dose decisions, and supportive-care use (4–6). Comparative peginterferon safety data in chronic viral hepatitis and CHB-specific myelosuppression-risk research further support the need for careful hematologic monitoring during treatment (7, 8).

Although hematologic decline during interferon therapy is clinically familiar, the relationship between longitudinal blood count changes, early serological response groups, and host baseline molecular state remains incompletely characterized in small prospective cohorts. Distinguishing a broad treatment-associated hematologic pattern from a pattern confined to a clinical response subgroup is important, particularly when the same cohort is also used for response-focused transcriptomic analyses.

Whole-blood RNA-seq provides a practical view of baseline host immune and hematologic state, but interpretation requires caution. Signals in whole blood may reflect both transcriptional programs and shifts in circulating cell composition (9, 10). Reference-based cell-composition estimates depend on the suitability of reference expression profiles and should be interpreted in light of relevant benchmarking data (11, 12). For that reason, transcriptomic findings in this setting require conservative interpretation and careful endpoint definition.

Because peginterferon-associated leukopenia, neutropenia, and thrombocytopenia are established adverse reactions, the purpose of this study was not to identify a new toxicity. Instead, we undertook an exploratory safety-phenotype analysis in a prospectively followed 21-patient chronic hepatitis B cohort to: (i) jointly quantify early WBC, ANC, and platelet trajectories and their uncertainty; (ii) examine whether the magnitude of decline differed by an operational early serological-response phenotype; (iii) evaluate how documented supportive-treatment timing affected interpretation of longitudinal counts; and (iv) reassess whole-blood transcriptomic associations with clinically prioritized hematologic endpoints using a DESeq2 variance-stabilizing-transformation workflow and complete multiplicity reporting. These analyses were intended to refine endpoint interpretation and generate testable hypotheses rather than establish a new adverse reaction, a predictive biomarker, or a causal mechanism.

## Materials and methods

### Study design, ethics, and cohort

This hematologic safety-focused analysis used the same prospective observational cohort as a related response-focused transcriptomic study but addressed a distinct clinical safety question and was organized according to transparent reporting principles for observational studies (13). Adults with chronic hepatitis B were prospectively enrolled at the Second Affiliated Hospital and Yuying Children's Hospital of Wenzhou Medical University between July 2024 and February 2025. Eligibility was defined according to the Guidelines for the Prevention and Treatment of Chronic Hepatitis B (version 2022) (14). The study was approved by the institutional ethics committee (Approval No. 2024-K-203-01; July 23, 2024), and all participants provided written informed consent.

The present study was an exploratory secondary analysis conducted at a single center. The cohort size was determined by the available parent cohort rather than by a confirmatory sample-size calculation, and no independent validation cohort was available. Accordingly, effect estimates and confidence intervals are reported to describe magnitude and uncertainty, while transcriptomic analyses are treated as hypothesis-generating.

The analysis included all 21 enrolled participants with baseline whole-blood RNA-seq data and an evaluable early-response classification. The main analysis set included 13 responders and 8 non-responders. P005 was retained in the clinical hematology cohort and in baseline transcriptomic analyses, but was excluded from analyses requiring paired visit-3/Week-10 RNA-seq data because the third RNA-seq specimen was missing.

Key exclusion criteria in the parent protocol included concomitant infections, other liver diseases or suspected hepatocellular carcinoma, Child-Pugh class B or C liver function, clinically important cardiovascular or cerebrovascular disease, immune-mediated disease, pregnancy or lactation, severe psychiatric disorders, marked cytopenia, severe renal dysfunction, and contraindications to interferon therapy.

### Treatment and response-group definition

All participants received peginterferon alfa-2b 180 μg subcutaneously once weekly, with or without background oral nucleos(t)ide analogue therapy. Peginterferon treatment continued beyond the Week-15 early-response assessment window.

Early serological response was operationally defined according to baseline HBsAg status. Among participants with detectable HBsAg at baseline, response was defined as either a ≥1.0 − log10 IU/mL (≥90%) decline from baseline at any scheduled assessment through Week 15 or a transition from detectable HBsAg to below the assay lower limit of detection (LLOD) by Week 15. Among participants whose HBsAg was already below the LLOD at baseline and whose anti-HBs was <10 mIU/mL, response was defined as maintenance of HBsAg below the LLOD together with anti-HBs seroconversion to ≥10 mIU/mL by Week 15. Participants meeting none of these criteria were classified as non-responders. P011 had HBsAg below the LLOD at baseline and throughout follow-up and was classified as a responder because anti-HBs increased from <2.00 mIU/mL at baseline to 11.29 mIU/mL at Week 15; this should not be described as treatment-emergent HBsAg loss, HBsAg seroclearance, or functional cure.

### Clinical hematology and supportive therapy

Serum HBsAg, anti-HBs, HBeAg, HBV DNA, ALT, AST, and routine hematologic variables were assessed at scheduled clinical visits in the parent cohort. For the present analysis, WBC, ANC, and platelet counts were evaluated longitudinally across admission-defined visits. Visit 2 occurred at a median of 34 days after visit 1 (IQR: 34–35 days; median week 4.9), and visit 3/R3 occurred at a median of 70 days after visit 1 (IQR: 66–70 days; median week 10.0). Observed nadirs and relative declines from baseline were calculated for descriptive clinical summaries. Relative decline was interpreted as a descriptive magnitude measure rather than as an untreated natural-history endpoint because supportive care was used after WBC decline.

Supportive-treatment timing was reconstructed in a deidentified patient-level event table. For oral leukocyte-support medication, each scheduled CBC was classified relative to the first documented treatment date: 0 denoted a CBC before that date and 1 denoted a CBC after that date. This variable is termed the post-documented oral-support-date indicator because the available records did not establish continuous active treatment, stop dates, adherence, or complete duration. Known within-day order was retained; visits with unresolved same-day order were omitted from the primary model and evaluated under both unexposed and exposed boundary assumptions. We additionally censored observations after the documented boundary and defined a strict Visit-1-to-Visit-3 paired sensitivity set in which both CBCs preceded the boundary. No G-CSF exposure was documented within planned Visits 1–3, and G-CSF was not entered into the WBC/ANC models. Because the documented oral agents were used for leukocyte support, they were not entered mechanically into platelet models. These analyses were designed to assess sensitivity to supportive-management timing, not to estimate treatment efficacy.

### Endpoint definitions

Primary clinical summaries included longitudinal WBC, ANC, and platelet trajectories from visit 1 to visit 3, observed nadirs, and relative declines from baseline. For transcriptomic association work, WBC <4.0 × 10^9^/L was retained only as a clinical-management context threshold rather than as a primary binary endpoint because it occurred in 19 of 21 patients.

Visit-2 platelet <100 × 10^9^/L and platelet relative decline were prioritized as the main exploratory platelet endpoints. ANC <1.0 × 10^9^/L and ANC relative decline were retained as secondary exploratory endpoints. WBC < 3.0 × 10^9^/L and WBC < 2.5 × 10^9^/L were retained as supplementary WBC endpoints.

### RNA-seq, preprocessing, and pathway scoring

Peripheral whole-blood RNA samples in the parent cohort were collected at Week 0, Week 5, and Week 10 using PAXgene Blood RNA Tubes. Total RNA was extracted using the PAXgene Blood miRNA Kit (QIAGEN; Cat. No. 763134) according to the manufacturer's instructions. RNA integrity was assessed using an Agilent 2100 Bioanalyzer before library preparation. RNA-seq libraries were prepared using the Fast RNA-seq Lib Prep Kit V2 (ABclonal; Cat. No. RK20306) and sequenced on an Illumina NovaSeq 6000 platform using paired-end 150-bp reads.

Raw sequencing reads were filtered using fastp v0.19.7. Clean reads were aligned to the Homo sapiens hg38 reference genome using HISAT2 v2.2.1 with default parameters. Gene-level read counts were generated using featureCounts v2.0.6. Reads with mapping quality below 10, reads not properly paired, and reads mapping to multiple genomic regions were excluded. The annotated gene-level raw-count matrix was used for downstream analyses.

For the present Paper 4 analysis, baseline whole-blood RNA-seq was available for all 21 patients and was used for baseline Hallmark pathway and candidate-gene association analyses. Paired visit-3/Week-10 RNA-seq was available for 20 patients and was used only for analyses requiring paired visit-3 transcriptomic data. Count data were normalized using the variance-stabilizing transformation in DESeq2 (15). GSVA was performed using the DESeq2 variance-stabilizing-transformation-normalized expression matrix and the Hallmark collection retrieved through msigdbr from MSigDB release 2026.1 (16, 17).

Baseline pathway activity was summarized using Hallmark GSVA scores. Associations between baseline pathway scores and early hematologic endpoints were evaluated as exploratory analyses. Baseline hematologic counts were evaluated as potential context variables because baseline WBC, ANC, and platelet values were associated with later absolute counts and nadirs. Analyses accounting for baseline counts were used as exploratory checks rather than confirmatory models.

Candidate genes within prioritized exploratory directions were evaluated for effect direction, expression reliability, and sensitivity to low-expression or zero-inflation concerns. Candidate-gene findings were assigned to supplementary interpretation unless they were direction-stable, expression-reliable, and aligned with a prioritized pathway-level direction.

### Statistical analysis

Within each response group, Visit-1-to-Visit-3 changes in WBC, ANC, and platelet count were evaluated using paired Wilcoxon signed-rank tests. We report the median paired change with IQR, the matched-pairs rank-biserial correlation, and patient-bootstrap percentile 95% confidence intervals based on 10,000 resamples. Between-group comparisons of observed nadirs and relative declines used Wilcoxon rank-sum tests. Effect sizes are reported in the responder-minus-non-responder direction as the Hodges–Lehmann location shift with its Wilcoxon 95% confidence interval and Cliff's delta with a response-stratified 10,000-resample bootstrap percentile 95% confidence interval. Binary endpoint frequencies are reported with exact Clopper-Pearson 95% confidence intervals. Bootstrap analyses used seed 20260920. No imputation or significance-based exclusion was performed, and all intervals are interpreted descriptively because of the small cohort.

For WBC and ANC, supportive-treatment timing was evaluated in an exploratory random-intercept model fitted by maximum likelihood: outcome ∼ visit × response group + post-documented oral-support date + (1|patient). The unadjusted comparator used the identical analysis rows without the support indicator. The primary boundary retained adjudicated within-day order and excluded visits with unresolved same-day order; two boundary analyses coded those unresolved visits as unexposed and exposed, respectively. We also repeated the longitudinal analysis after censoring post-boundary observations and performed a strict Visit-1-to-Visit-3 paired pre-support analysis. Wald 95% confidence intervals and unadjusted normal-approximation P values are reported for transparency. Model rank, convergence, and singularity were checked. Because support was initiated in response to low counts and active treatment intervals were unavailable, the support coefficient was not interpreted causally.

Hallmark and candidate-gene analyses used complete defined multiple-testing families with BH-FDR correction. In the unadjusted primary VST analyses, no Hallmark or candidate-gene result met the prespecified exploratory *q* < 0.10 threshold. Cell-composition sensitivity analysis used xCell 2.0 (18). Cell-type enrichment was estimated from the TPM matrix for all 62 RNA samples using xCell2 v1.2.3. ImmuneCompendium.xCell2Ref was the primary human RNA-seq immune/blood reference and BlueprintEncode.xCell2Ref was used as a reference sensitivity analysis. Scores were generated with spillover correction (rawScores = FALSE, spillover = TRUE, spilloverAlpha = 0.5, minSharedGenes = 0.85) and interpreted as relative enrichment scores, not measured cell fractions, proportions, or absolute counts. The primary-reference neutrophil score was selected *a priori* and assessed for internal concordance with matched ANC and WBC using Spearman correlations with patient-level or patient-cluster bootstrap 95% confidence intervals based on 2,000 resamples. Baseline Hallmark associations were evaluated using M0 (pathway), M1 (pathway plus the corresponding baseline blood count), and M2 (M1 plus standardized baseline neutrophil enrichment). A reviewer-directed paired sensitivity analysis in the 20 patients with W0 and W10 RNA used W10-minus-W0 Hallmark-score changes and added the corresponding neutrophil-score change in M2. Continuous endpoints used rank-linear models and binary endpoints used Firth logistic regression. BH q values were calculated separately within each complete endpoint-by-model family of 50 Hallmark pathways. Models with nonconverged profile-likelihood intervals were retained and flagged but not interpreted.

Analyses were performed using R v4.5.2. Key R/Bioconductor packages included DESeq2 v1.50.2, GSVA v2.4.9, msigdbr v26.1.0, SummarizedExperiment v1.40.0, lme4 v2.0-1, xCell2 v1.2.3, boot v1.3-32, tidyverse v2.0.0, ggplot2 v4.0.3, openxlsx v4.2.8.1, svglite v2.2.2, and related plotting and data-handling packages.

### Sensitivity analyses

Because P011 entered the cohort with HBsAg already below the assay LLOD and met the anti-HBs seroconversion component of the operational response definition, we conducted an internal sensitivity analysis excluding P011 without reassigning the participant to the non-responder group. The primary cohort remained 13 responders and 8 non-responders; the exclusion sensitivity included 12 responders and 8 non-responders and was used only to assess direction-level robustness.

We retained all 21 participants (13 responders and 8 non-responders) in the primary clinical analysis. We also repeated the clinical sensitivity analysis excluding the sole participant with positive baseline HBeAg (P021), leaving 13 responders and 7 non-responders. Within-group changes from Visit 1 to Visit 3 were assessed using two-sided Wilcoxon signed-rank tests; between-group nadir and relative-decline comparisons used two-sided Wilcoxon rank-sum tests. Asymptotic P values with continuity correction were used (exact = FALSE, correct = TRUE). These nominal sensitivity P values were interpreted descriptively (Supplementary Table S19).

## Results

### Cohort structure, visit timing, and analysis inclusion

The study included 21 patients with chronic hepatitis B receiving peginterferon alfa-2b-based therapy, comprising 13 responders and 8 non-responders. Clinical hematology data were retained for all 21 patients. Admission-defined visit timing placed visit 2 at a median of 34 days after visit 1 (IQR: 34–35 days; median week 4.9) and visit 3/R3 at a median of 70 days after visit 1 (IQR: 66–70 days; median week 10.0). Baseline RNA-seq was available for all 21 patients, whereas paired visit-3/Week-10 RNA-seq was available for 20 patients because P005 lacked the third transcriptomic specimen. P005 was therefore retained in the clinical hematology cohort and baseline transcriptomic analyses, but excluded from analyses requiring paired visit-3 transcriptomic data. The corrected platelet value series for P004 was used in this clinical analysis, supporting an observed platelet nadir of 89 × 10^9^/L rather than the historical erroneous value.

These cohort, timing, and assay-specific availability boundaries are summarized in Figure 1.

**Figure 1:**
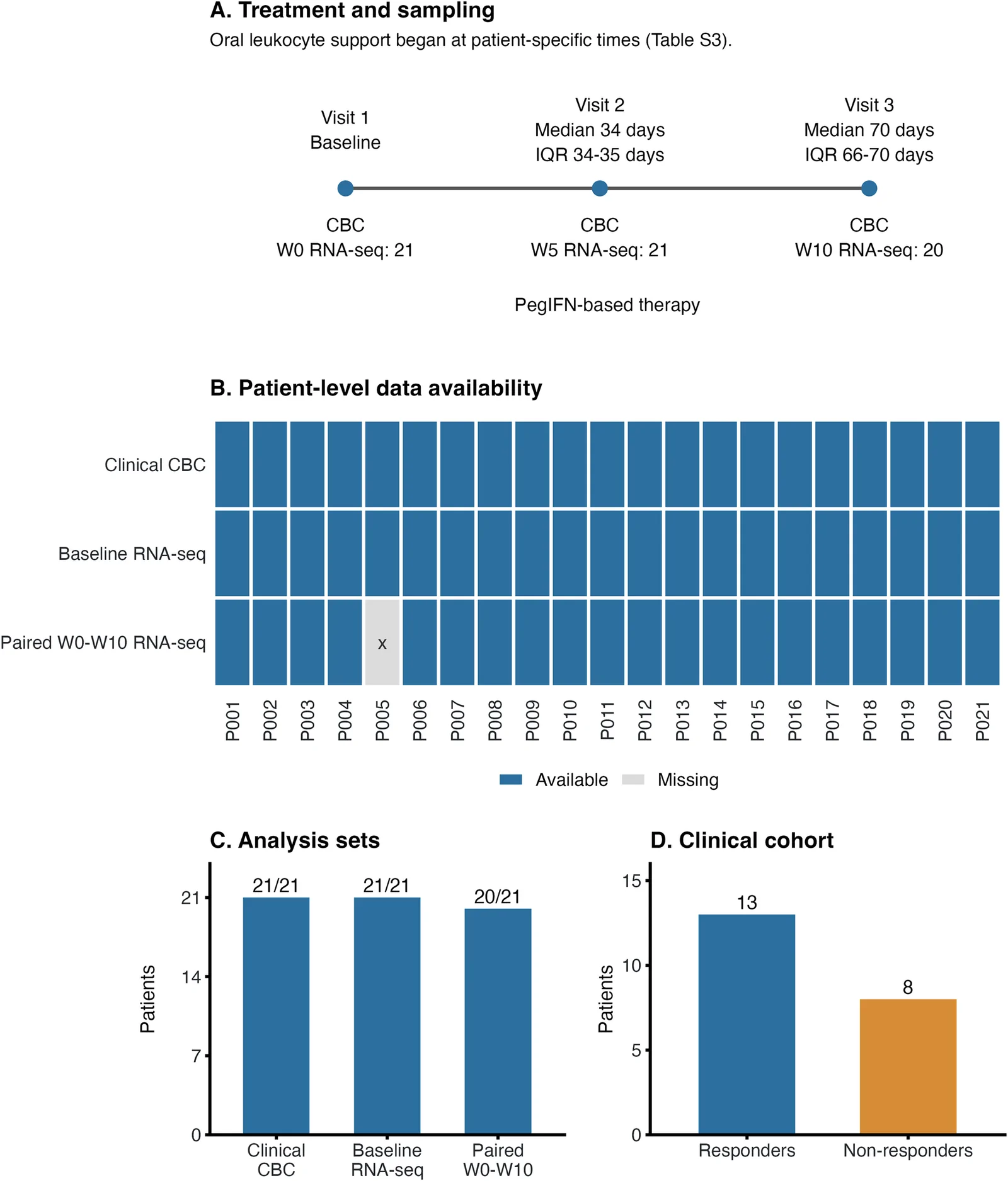
Study design, supportive-care context, and data availability. **(A)** Scheduled CBC and RNA-seq assessments during PegIFN-based therapy. Visit timing is expressed relative to baseline. Oral leukocyte support began at patient-specific times; the timeline does not imply a uniform prescribing rule or a common Visit-2 start. Patient-level treatment timing is provided in Supplementary Table S3, and sensitivity analyses are summarized in Figure 3. **(B)** Patient-level availability of clinical CBC, baseline RNA-seq, and paired W0–W10 RNA-seq. The cross denotes the missing P005 W10 RNA sample. **(C)** Available patients in each analysis set. **(D)** The clinical cohort comprised 13 responders and 8 non-responders. All 21 patients remained in the main clinical cohort. RNA-seq included 62 samples (W0/W5/W10: 21/21/20).

### Peginterferon alfa-2b-based therapy was associated with longitudinal Wbc, Anc, and platelet decline

WBC, ANC, and platelet counts declined from Visit 1 to Visit 3 in both response groups (Figure 2; Main Tables 1–2; Supplementary Table S1). Among responders (*n* = 13), the median paired changes were −2.430 × 10^9^/L for WBC (bootstrap 95% CI: −2.530 to −1.530; *P* = 0.00166), −1.730 × 10^9^/L for ANC (95% CI: −1.990 to −1.094; *P* = 0.00166), and −67.0 × 10^9^/L for platelets (95% CI: −83.0 to −55.0; *P* = 0.00166). Among non-responders (*n* = 8), the corresponding changes were −2.745 × 10^9^/L for WBC (95% CI: −3.570 to −2.190; *P* = 0.0143), −1.870 × 10^9^/L for ANC (95% CI: −2.260 to −1.110; *P* = 0.0143), and −97.5 × 10^9^/L for platelets (95% CI: −112.0 to −64.0; *P* = 0.0143). Every observed paired change was negative; the matched-pairs rank-biserial correlation was −1.00 in each group for each measure. The IQRs and bootstrap intervals for both paired effect-size summaries are reported in Supplementary Table S1.

**Figure 2:**
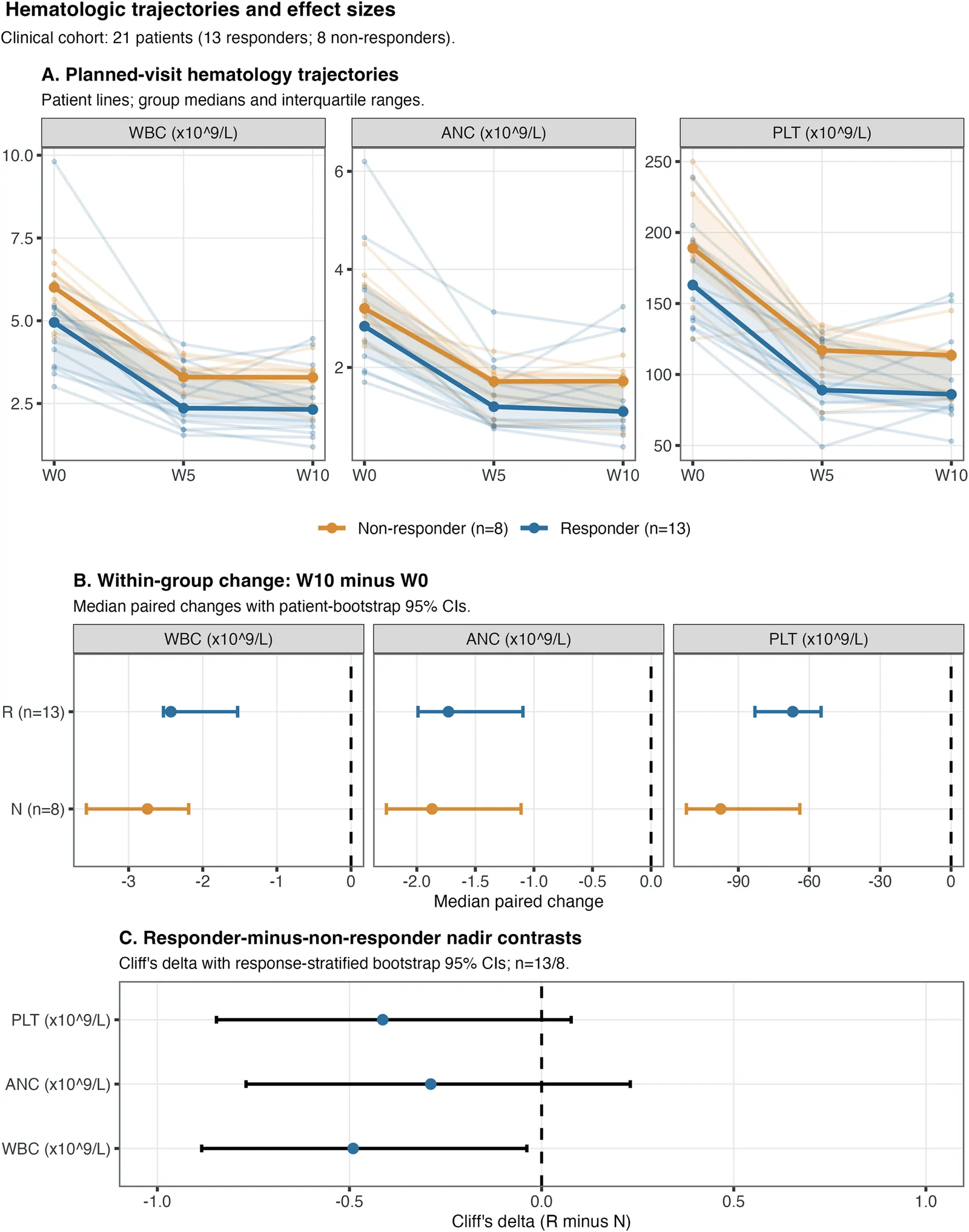
Longitudinal hematologic changes and effect-size summaries. Longitudinal WBC, ANC, and platelet changes are shown for the 21-patient cohort (13 responders and 8 non-responders). **(A)** Thin lines show individual patients, thick lines show group medians, and ribbons show interquartile ranges across Weeks 0, 5, and 10. **(B)** Median paired Week-10-minus-Week-0 changes with patient-bootstrap 95% confidence intervals. **(C)** Responder-minus-non-responder observed-nadir contrasts are shown as Cliff's delta with response-stratified bootstrap 95% confidence intervals. Effect sizes and confidence intervals are descriptive because of the small, single-center cohort.

**Table 1:** Baseline hematologic values and observed nadirs by response group.

Response group	Patients, n	Baseline WBC, median (Q1–Q3), ×10^9^/L	Baseline ANC, median (Q1–Q3), ×10^9^/L	Baseline platelet count, median (Q1–Q3), ×10^9^/L	Observed WBC nadir, median, ×10^9^/L	Observed ANC nadir, median, ×10^9^/L	Observed platelet nadir, median, ×10^9^/L
Non-responder	8	6.01 (4.89-6.48)	3.21 (2.82-3.74)	189.0 (172.5-229.8)	3.25	1.69	112.0
Responder	13	4.95 (3.62-5.41)	2.84 (2.23-3.58)	163.0 (138.0-193.0)	2.29	1.10	86.0

Table 1 summarizes baseline WBC, ANC, and platelet values together with observed hematologic nadirs by early response group. Continuous variables are shown as median (Q1–Q3).

**Table 2:** Hematologic endpoints and sensitivity analyses.

**Analysis**	**Endpoint/group**	**Estimate**	**95% CI or complementary effect**	**P value**
Paired V1-V3	WBC, R (*n* = 13)	Median change: −2.430	Bootstrap CI: −2.530 to −1.530; rank-biserial −1.00	0.00166
Paired V1-V3	WBC, N (*n* = 8)	Median change: −2.745	Bootstrap CI: −3.570 to −2.190; rank-biserial −1.00	0.014
Paired V1-V3	ANC, R (*n* = 13)	Median change: −1.730	Bootstrap CI: −1.990 to −1.094; rank-biserial −1.00	0.00166
Paired V1-V3	ANC, N (*n* = 8)	Median change: −1.870	Bootstrap CI: −2.260 to −1.110; rank-biserial −1.00	0.014
Paired V1-V3	PLT, R (*n* = 13)	Median change: −67.0	Bootstrap CI: −83.0 to −55.0; rank-biserial −1.00	0.00166
Paired V1-V3	PLT, N (*n* = 8)	Median change: −97.5	Bootstrap CI: −112.0 to −64.0; rank-biserial −1.00	0.014
Between-group nadir	WBC, *R* − *N*	HL shift: −0.810	HL CI: −1.240 to 0.160; Cliff's delta: −0.490 (−0.885 to −0.038)	0.070
Between-group nadir	ANC, *R* − *N*	HL shift: −0.391	HL CI: −0.830 to 0.330; Cliff's delta: −0.288 (−0.769 to 0.231)	0.294
Between-group nadir	PLT, *R* − *N*	HL shift: −20.0	HL CI: −39.0 to 7.0; Cliff's delta: −0.413 (−0.846 to 0.077)	0.128
Binary endpoint	Visit-2 WBC <4.0 × 10^9^/L	19/21 (90.5%)	Exact CI: 69.6% to 98.8%	Descriptive
Binary endpoint	Visit-2 ANC <1.0 × 10^9^/L	7/21 (33.3%)	Exact CI: 14.6% to 57.0%	Descriptive
Binary endpoint	Visit-2 PLT <100 × 10^9^/L	10/21 (47.6%)	Exact CI: 25.7% to 70.2%	Descriptive
Support-timing sensitivity	WBC, post-date term	−0.008 × 10^9^/L	−0.637 to 0.620; 60 observations/21 patients	0.979
Support-timing sensitivity	ANC, post-date term	−0.105 × 10^9^/L	−0.536 to 0.326; 60 observations/21 patients	0.632
Strict pre-support sensitivity	WBC and ANC V1-V3, R = 5/*N* = 4	WBC median change R: −2.480, N: −3.035; ANC R: −1.890, N: −1.980	Both scheduled CBCs before the documented support boundary	R 0.059; N 0.100

Table 2 summarizes paired Visit-1-to-Visit-3 effects, responder-minus-non-responder nadir effects, exact Visit-2 endpoint frequencies, the non-causal support-timing model term, and the strict pre-support paired sensitivity set.

Abbreviations: V1, Visit 1; V3, Visit 3; ANC, absolute neutrophil count; WBC, white blood cell count; PLT, platelet count; HL, Hodges–Lehmann. WBC, ANC, and PLT values are ×10^9^/L. Paired effects are V3 − V1 and include 10,000-resample patient-bootstrap confidence intervals and matched-pairs rank-biserial correlations. Between-group effects are responder minus non-responder and include HL shifts and Cliff's delta. Binary intervals are exact Clopper-Pearson intervals. The post-date coefficient is a non-causal sensitivity estimate; strict pre-support *R* = 5/*N* = 4 does not replace the primary 13*R*/8*N* cohort.

Between-group estimates were imprecise and did not establish equivalence or a response-group-specific toxicity pattern. For observed nadirs, the responder-minus-non-responder Hodges–Lehmann shifts were −0.391 × 10^9^/L for ANC (95% CI: −0.830 to 0.330; Cliff's delta: −0.288, bootstrap 95% CI: −0.769 to 0.231; *P* = 0.294), −0.810 × 10^9^/L for WBC (95% CI: −1.240 to 0.160; Cliff's delta: −0.490, bootstrap 95% CI: −0.885 to −0.038; *P* = 0.070), and −20.0 × 10^9^/L for platelets (95% CI: −39.0 to 7.0; Cliff's delta: −0.413, bootstrap 95% CI: −0.846 to 0.077; *P* = 0.128). Relative-decline comparisons were also imprecise: ANC shift 0.0369 (95% CI: −0.0862 to 0.1519; Cliff's delta: 0.250, bootstrap 95% CI: −0.308 to 0.731; *P* = 0.365), WBC shift 0.0143 (95% CI: −0.0621 to 0.1119; Cliff's delta: 0.0577, bootstrap 95% CI: −0.462 to 0.558; *P* = 0.856), and platelet shift −0.0143 (95% CI: −0.0825 to 0.1046; Cliff's delta: −0.135, bootstrap 95% CI: −0.654 to 0.385; *P* = 0.638).

After excluding the sole baseline HBeAg-positive participant, WBC, ANC, and platelet counts still decreased from Visit 1 to Visit 3 in both response groups (all paired *P* = 0.002 in responders and *P* = 0.022 in the seven remaining non-responders). Between-group comparisons of observed nadirs (*P* = 0.104–0.383) and relative declines (*P* = 0.383–0.874) did not provide strong evidence of a response-group difference (Supplementary Table S19). These small-sample sensitivity findings do not establish equivalence between groups.

### WBC <4.0 × 10^9^/L provided clinical-management context but was not a discriminating endpoint

WBC <4.0 × 10^9^/L was treated as a pragmatic clinical-management context threshold rather than as a primary transcriptomic binary endpoint (Figure 3; Main Table 2; Supplementary Tables S2, S3). At Visit 2, WBC <4.0 × 10^9^/L occurred in 19/21 patients (90.5%; exact 95% CI: 69.6%–98.8%), ANC <1.0 × 10^9^/L in 7/21 (33.3%; 95% CI: 14.6%–57.0%), and platelet count <100 × 10^9^/L in 10/21 (47.6%; 95% CI: 25.7%–70.2%). The near-universal WBC <4.0 × 10^9^/L event rate limited its value as a discriminating transcriptomic endpoint in this cohort.

**Figure 3:**
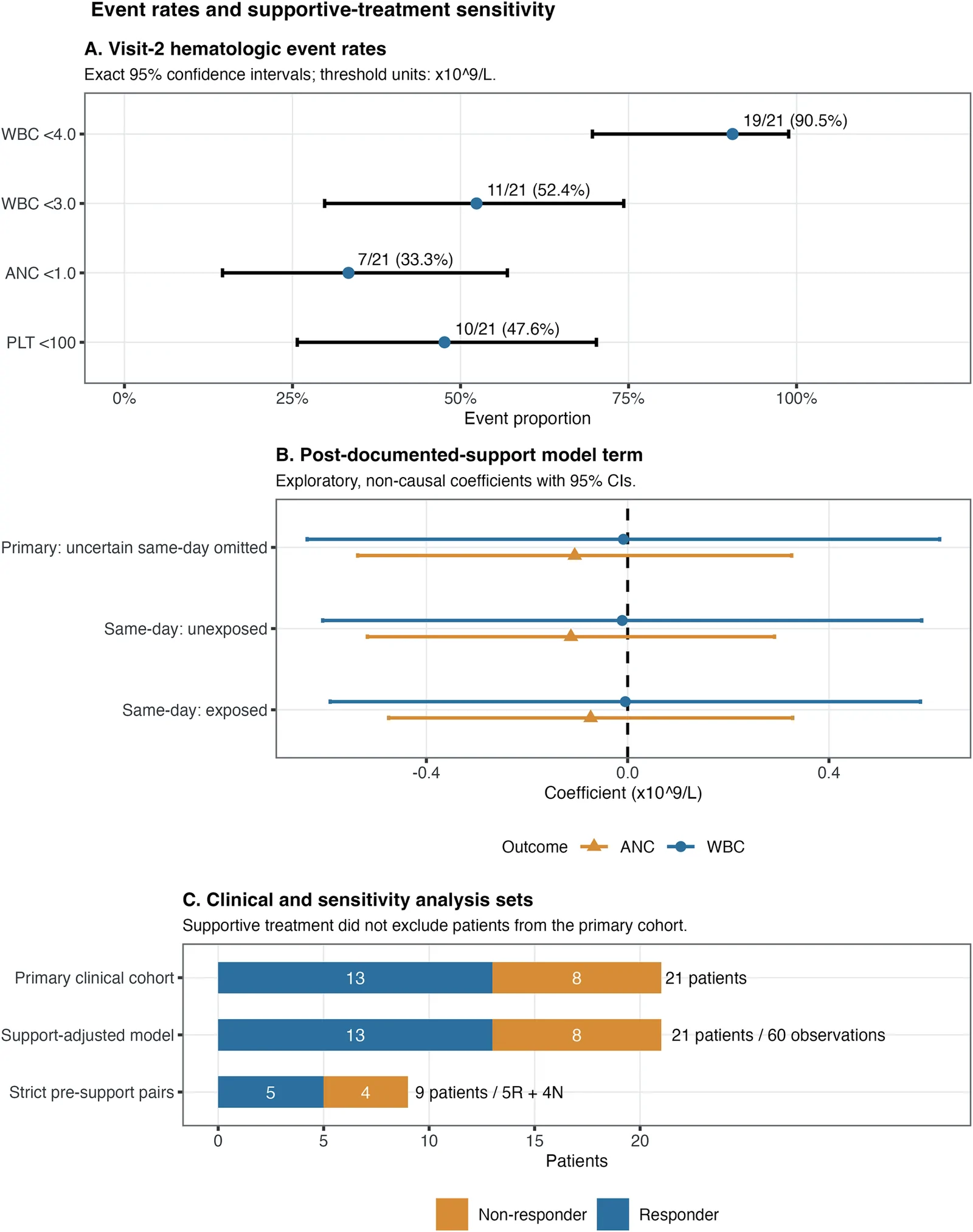
Hematologic event rates and supportive-treatment sensitivity analyses. **(A)** Visit-2 event proportions for WBC <4.0 × 10^9^/L, WBC <3.0 × 10^9^/L, ANC <1.0 × 10^9^/L, and platelet count <100 × 10^9^/L, with exact Clopper-Pearson 95% confidence intervals. The source-verified P005 Visit-2 ANC value was 0.75 × 10^9^/L; accordingly, ANC <1.0 × 10^9^/L occurred in 7/21 participants. **(B)** Post-documented-oral-support coefficients and 95% confidence intervals for WBC and ANC under the primary analysis and two same-day boundary assumptions. These coefficients are exploratory and non-causal. **(C)** Analysis-set context: supportive treatment did not remove participants from the 21-patient primary cohort; the primary support-adjusted model included 60 observations from all 21 participants, whereas the strict pre-support Week-0-to-Week-10 paired sensitivity set included nine participants (five responders and four non-responders). Confounding by indication, reverse causality, unknown stop dates, and unresolved same-day timing preclude causal interpretation.

The primary support-adjusted WBC and ANC models included 60 planned-visit observations from all 21 participants: 43 observations before and 17 after the documented oral-support boundary, with 12 participants changing exposure state. Three observations with unresolved same-day ordering were omitted from the primary model. All adjusted and matched unadjusted fits were full rank, converged, and non-singular. The non-causal post-date estimate was −0.008 × 10^9^/L for WBC (95% CI: −0.637 to 0.620; *P* = 0.979) and −0.105 × 10^9^/L for ANC (95% CI: −0.536 to 0.326; *P* = 0.632). Coding the unresolved same-day visits as all unexposed or all exposed did not materially change these estimates or the fitted longitudinal pattern. Full timing and model outputs are provided in Supplementary Tables S3, S4.

After censoring observations following the documented support boundary, 43 observations from all 21 participants remained available across visits. The stricter Visit-1-to-Visit-3 paired set retained five responders and four non-responders. All median paired changes remained negative: WBC −2.480 × 10^9^/L in responders and −3.035 × 10^9^/L in non-responders, and ANC −1.890 × 10^9^/L in responders and −1.980 × 10^9^/L in non-responders (*P* = 0.059 for responders and *P* = 0.100 for non-responders). These small-set estimates are directionally consistent with decline before documented support, but their precision is insufficient for a confirmatory subgroup conclusion. Because treatment was initiated in response to low counts and active-treatment duration and adherence were incompletely observed, none of these analyses estimates supportive-treatment efficacy or removes confounding by indication. The strict paired results are provided in Supplementary Table S5.

For exploratory baseline transcriptomic analyses, platelet endpoints were prioritized because they were clinically relevant and more informative than the near-universal WBC < 4.0 threshold. Visit-2 platelet <100 × 10^9^/L and platelet relative decline were treated as the main exploratory platelet endpoints. ANC < 1.0 × 10^9^/L and ANC relative decline were retained as secondary exploratory endpoints. Baseline WBC, ANC, and platelet values were associated with later absolute count endpoints and nadirs, supporting the importance of baseline counts when interpreting endpoints. Relative-decline endpoints were less directly explained by baseline counts.

### Unadjusted primary VST analyses produced no associations at *q* < 0.10

In the baseline VST-based Hallmark analyses, none of the 350 associations across seven endpoint-specific families met the exploratory BH-FDR threshold of *q* < 0.10 (minimum *q* = 0.251). WBC <4.0 × 10^9^/L remained clinical-management context rather than a primary discriminating transcriptomic endpoint. Figure 4A summarizes the minimum nominal P value in each family, and Figure 4B displays the ten highest and ten lowest correlations with Visit-2 platelet relative decline; this rank-based display retains *q* values calculated over all 50 Hallmark pathways in the corresponding endpoint family. EMT, which was highlighted in the original submission, is shown for continuity in Figures 4C–D rather than as a newly prioritized pathway. Its association with platelet relative decline was positive but imprecise (rho = 0.384, 95% CI: −0.059 to 0.707; nominal *P* = 0.085, *q* = 0.555). Baseline EMT scores differed between patients with and without Visit-2 platelet counts <100 × 10^9^/L (median difference = 0.144; nominal *P* = 0.022, *q* = 0.264), but this association also did not meet the FDR threshold. Complete baseline results are provided in Supplementary Table S6. Paired W10-minus-W0 analyses are reported separately in Supplementary Figure S2 and Table S8; none of the 150 paired Hallmark associations met *q* < 0.10 (minimum *q* = 0.671). All pathway findings remain exploratory.

**Figure 4:**
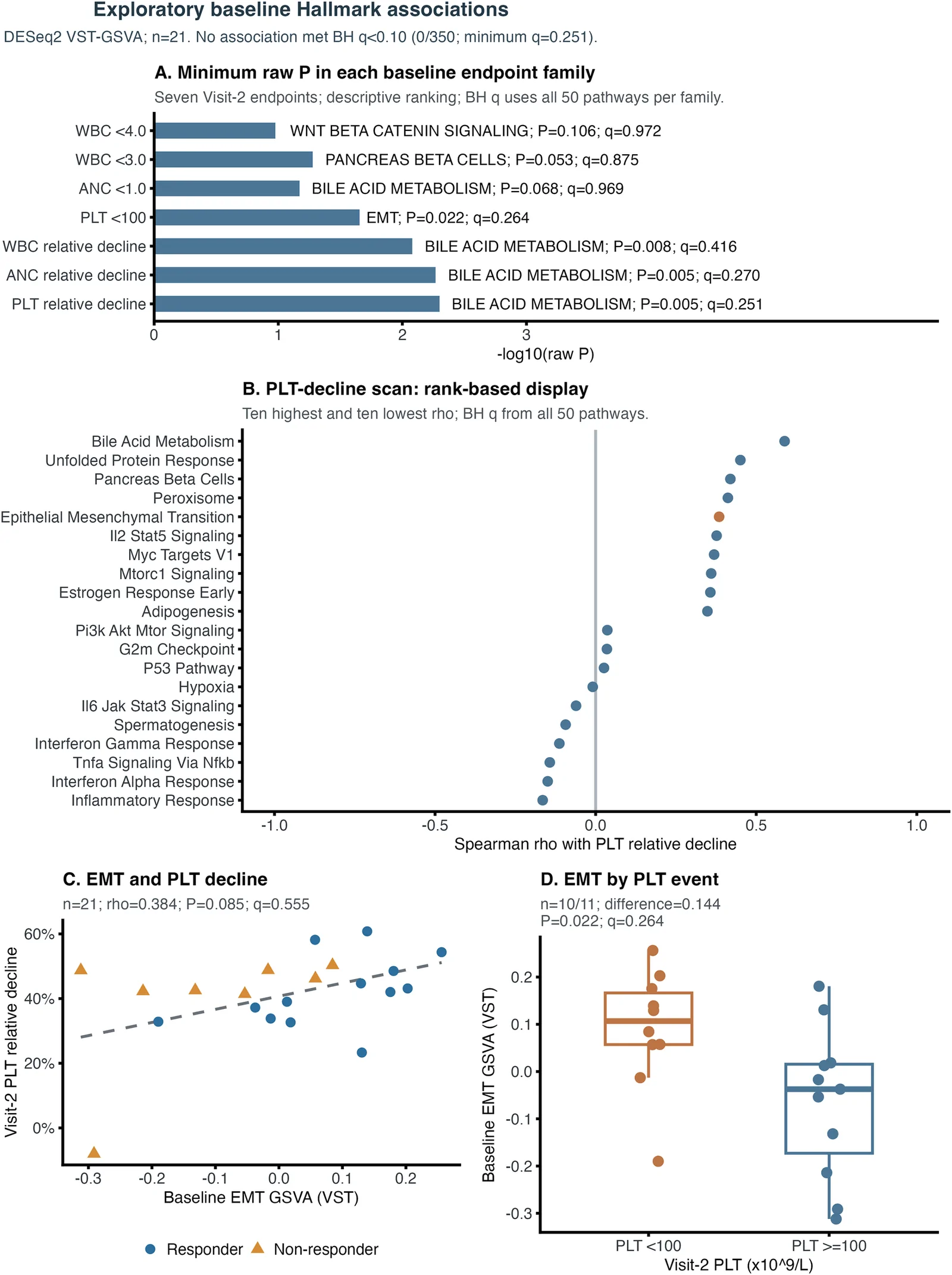
Exploratory baseline VST-based Hallmark associations with hematologic endpoints. Hallmark GSVA scores were calculated from the DESeq2 VST (blind = FALSE) expression matrix. **(A)** Minimum nominal P value among all 50 Hallmark pathways for each of the seven baseline-to-Visit-2 endpoint families; pathway names, nominal P values, and BH-adjusted q values are shown. WBC <4.0 × 10^9^/L is included as clinical-management context, not as a primary discriminating transcriptomic endpoint. This is a descriptive ranking, not confirmatory evidence. **(B)** The ten highest and ten lowest Spearman correlations between baseline Hallmark scores and Visit-2 platelet relative decline, selected by correlation rank for display only. The orange point denotes EMT. BH q values are retained from the complete 50-pathway endpoint family, without recalculation on the displayed subset; complete effect estimates, nominal P values, and q values are provided in Supplementary Table S6. **(C)** Baseline EMT Hallmark score versus Visit-2 platelet relative decline in 21 patients (Spearman rho = 0.384, nominal *P* = 0.085, BH *q* = 0.555). The dashed line is a descriptive linear guide and is not a Spearman fit. **(D)** Baseline EMT scores by Visit-2 platelet count <100 × 10^9^/L (10 vs. 11 patients; event-minus-no-event median difference = 0.144, Wilcoxon *P* = 0.022, BH *q* = 0.264). Box plots show the median and interquartile range, whiskers extend to the most extreme values within 1.5 interquartile ranges, and all patient values are shown. EMT is displayed because it was highlighted in the original submission, not because it was selected as the strongest revised signal. No baseline Hallmark association met *q* < 0.10; all analyses are exploratory. Paired analyses are reported separately in Supplementary Figure S2 and Table S8.

No baseline candidate-gene association met *q* < 0.10 (minimum *q* = 0.417). For transparency, the VST-based SPP1 contrast for Visit-2 platelet <100 × 10^9^/L had an event-minus-non-event median difference of −0.498 (*P* = 0.00435, *q* = 0.417), whereas SAT1 versus platelet relative decline had rho = −0.313 (*P* = 0.167, *q* = 0.883). Neither result was prioritized as a biomarker or mechanism (Supplementary Figure S1 and Table S7).

### Supplementary paired VST transcriptomic analysis

Among the 20 patients with paired W0 and W10 RNA-seq, no delta Hallmark or six-gene candidate association met *q* < 0.10 (minimum *q* = 0.671 and 0.117, respectively). The top-ranked Visit-3 platelet <100 × 10^9^/L Hallmark contrast was APICAL SURFACE (difference = −0.199, *P* = 0.0227, *q* = 0.671), and delta SPP1 versus platelet relative decline had rho = −0.311 (*P* = 0.182, *q* = 0.676). P005 was excluded only from paired RNA analyses because W10 RNA was unavailable. These results remain supplementary and hypothesis-generating (Supplementary Figure S2 and Tables S8, S9).

### xCell2 cell-composition sensitivity analysis

The primary-reference neutrophil enrichment score showed modest and imprecise baseline concordance with ANC (Spearman rho = 0.382; bootstrap 95% CI: −0.110 to 0.732) and WBC (rho = 0.122; 95% CI: −0.344 to 0.572), but stronger positive concordance across matched visits (ANC rho = 0.693, patient-cluster bootstrap 95% CI: 0.448–0.856; WBC rho = 0.559, 95% CI: 0.314–0.754) and for W0-W10 changes (ANC rho = 0.646, 95% CI: 0.343–0.840; WBC rho = 0.556, 95% CI: 0.172–0.806). No unadjusted baseline Hallmark family and no paired W0-W10 M0/M1/M2 family contained a pathway with within-family *q* < 0.10. For the baseline platelet <100 × 10^9^/L endpoint, 3/50 M1 and 4/50 M2 pathways reached *q* < 0.10, but none reached *q* < 0.05; four of these seven rows had nonconverged profile-likelihood intervals and the remaining intervals were extremely wide. These adjusted platelet signals were retained in the Supplement as unstable exploratory results and were not used for pathway prioritization or mechanistic inference (Supplementary Figure S3 and Tables S10–S18).

## Discussion

Leukopenia, neutropenia, and thrombocytopenia are established adverse reactions of interferon-based treatment and are not novel observations of this study (4–8). The added value of this exploratory cohort is narrower: WBC, ANC, and platelet trajectories were characterized synchronously within the same prospectively followed CHB population; the magnitude and uncertainty of change were quantified; response-group specificity was examined without treating non-significance as equivalence; and supportive-treatment timing was incorporated into sensitivity analyses rather than retained only as background information. The data show uniform within-group decline, but the wide between-group intervals preclude a precise claim about whether response phenotype modifies hematologic toxicity.

A second practical contribution concerns endpoint interpretation. Visit-2 WBC <4.0 × 10^9^/L occurred in 19/21 participants and therefore provided clinical-management context rather than an informative discriminating molecular endpoint in this cohort. The supportive-treatment sensitivity analyses did not materially change the fitted WBC or ANC pattern, but they also cannot isolate a treatment effect because support was initiated in response to low counts. Thus, later counts should be interpreted as hematologic measurements obtained during continued peginterferon therapy and routine clinical management, rather than as the untreated course of cytopenia (Supplementary Tables S3–S5). The VST-based transcriptomic reassessment and complete FDR reporting further delimit which molecular observations may be used for hypothesis generation; they do not validate a biomarker or establish clinical utility.

Several non-mutually-exclusive mechanisms provide biological context for interferon-associated cytopenia. Type-I-interferon receptors and signaling have been demonstrated in CD34+ hematopoietic progenitor cells (19), and clinical standard or pegylated interferon exposure can rapidly suppress hematopoiesis (20), supporting a plausible marrow-production component for leukocyte and neutrophil decline. In pegylated-interferon/ribavirin-treated chronic hepatitis C, serum G-CSF did not rise despite declining neutrophil counts; this was interpreted as an inadequate endogenous response (21). However, that observation arose in a different disease and treatment context, and we did not measure G-CSF. For platelets, experimental type-I-interferon signaling can reduce platelet production and function (22). A small study of conventional interferon in CHB suggested impaired platelet production and altered platelet distribution (5). In another chronic viral hepatitis study, antiplatelet-antibody positivity did not increase during treatment and was not associated with the decline in platelet counts (23). These heterogeneous findings provide mechanistic context but do not identify the mechanism operating in our cohort.

The evidence directly generated by this study is limited to the observed clinical and molecular measurements. WBC, ANC, and platelet counts declined from Visit 1 to Visit 3 in both response groups, but we did not measure bone-marrow function, progenitor-cell activity, endogenous G-CSF, antiplatelet antibodies, thrombopoiesis, or cell-intrinsic signaling. Moreover, no unadjusted primary VST Hallmark or candidate-gene family met the prespecified exploratory BH-FDR *q* < 0.10 threshold. Whole-blood expression also combines cell-intrinsic transcription with variation in circulating cell composition (9, 10). The xCell2 sensitivity analysis was consistent with an important cell-composition component in whole-blood expression because inferred neutrophil enrichment tracked measured ANC and WBC longitudinally and composition adjustment changed multiple pathway estimates. It did not establish a stable FDR-controlled within-person pathway association, and the unstable baseline-adjusted platelet signals were not interpreted as pathway discoveries. Consequently, pathway and candidate-gene observations—including EMT-, SPP1-, or SAT1-related directions—must remain exploratory and cannot be used as proof of mechanism.

The unadjusted primary VST reassessment materially changed the ranking of historical raw-level signals, and no Hallmark or candidate-gene family met *q* < 0.10. EMT-, SPP1-, SAT1-, APICAL SURFACE-, and other raw-P-ranked observations are therefore reported only to document the complete exploratory analysis. They do not establish a mechanism, biomarker, prediction model, or liver-intrinsic process.

This safety-oriented analysis used the same prospective cohort and RNA-seq resource as a companion response-focused transcriptomic study, but addressed a distinct clinical question: longitudinal hematologic decline, endpoint definition for WBC/ANC/platelet outcomes, and exploratory baseline whole-blood transcriptomic associations with hematologic endpoints.

Several features strengthen the clinical interpretation. The study uses admission-defined visits with explicit visit-timing summaries, and it retains all 21 patients for clinical hematology while applying assay-specific missingness rules for RNA-seq availability. The endpoint strategy also keeps the analysis clinically interpretable by treating WBC < 4.0 × 10^9^/L as a management threshold rather than forcing it into an uninformative primary transcriptomic endpoint. Importantly, the manuscript keeps the stronger clinical evidence separate from the weaker molecular signals instead of overstating the transcriptomic findings.

This study has substantial limitations. It is an exploratory secondary analysis of a small, single-center cohort of 21 participants, with no independent validation cohort. The wide confidence intervals around the between-group estimates do not establish equivalence and restrict generalization beyond the studied population and clinical setting. Because only one participant had positive baseline HBeAg, the 20-participant P021-exclusion sensitivity remains descriptive, does not alter the primary 21-participant cohort, and cannot support generalization to HBeAg-positive populations (Supplementary Table S19). Supportive treatment was not randomized: the post-documented-date indicator does not establish continuous active exposure, stop dates, dose, or adherence, and confounding by indication and reverse causality remain. The strict Visit-1-to-Visit-3 pre-support comparison included only five responders and four non-responders and was therefore imprecise. xCell2 produced relative enrichment scores rather than measured cell fractions; the score was inferred from the same bulk RNA data as the pathway scores, baseline concordance was imprecise, and only one prespecified leukocyte score was modeled to limit overfitting in *n* = 21. Residual composition confounding, mathematical coupling, and possible overadjustment therefore remain, and candidate-gene observations were not presented as composition-adjusted findings. Whole-blood transcriptomics cannot be interpreted as liver-intrinsic biology, and no unadjusted primary VST Hallmark or candidate-gene family met the prespecified exploratory BH-FDR *q* < 0.10 threshold. The molecular findings are therefore hypothesis-generating and require validation in larger, independent, preferably multicenter cohorts with orthogonal cell-composition and hematopoietic measurements.

In this exploratory 21-patient chronic hepatitis B cohort, WBC, ANC, and platelet counts declined during peginterferon alfa-2b-based therapy in both early response groups. Effect estimates were consistent with substantial within-group decline, whereas between-group estimates were imprecise and did not establish either response-group specificity or equivalence. Visit-2 WBC <4.0 × 10^9^/L was near-universal and was more informative as clinical-management context than as a discriminating transcriptomic endpoint. Timing-based supportive-treatment analyses did not materially change the fitted WBC or ANC pattern but cannot support causal treatment-effect conclusions. The VST-based whole-blood transcriptomic results did not provide FDR-supported mechanistic evidence. These findings should be considered hypothesis-generating and require confirmation in larger independent cohorts.

## Data Availability

The original contributions presented in the study are publicly available. This data can be found here: https://www.ncbi.nlm.nih.gov/geo/query/acc.cgi?acc=GSE334869 (accession number: GSE334869).
