# Supplementary material for: Longitudinal hematologic decline during peginterferon alfa-2b therapy in chronic hepatitis B: clinical safety and exploratory whole-blood transcriptomics
Source: Front Med (Lausanne). 2026 Oct 26;13:1919392. doi: 10.3389/fmed.2026.1919392 (PMC13746126; doi:10.3389/fmed.2026.1919392)
Supplement: Supplementary file 1 [file Datasheet2.docx]

Supplementary Figure and Table Legends

**Supplementary Figure S1. Baseline VST-based candidate-gene results for platelet endpoints.** Baseline expression was derived from the DESeq2 VST (blind=FALSE) symbol-mean matrix. **(A)** SPP1 by Visit-2 platelet count <100 ×10⁹/L (10 vs 11 patients; event-minus-no-event median difference=-0.498, Wilcoxon P=0.00435, BH q=0.417). **(B)** SPP1 versus Visit-2 platelet relative decline (Spearman rho=-0.194, patient-bootstrap 95% CI, -0.675 to 0.319; P=0.401, q=0.883). **(C)** SAT1 by Visit-2 platelet count <100 ×10⁹/L (10 vs 11 patients; event-minus-no-event median difference=-0.286, Wilcoxon P=0.245, q=0.785). **(D)** SAT1 versus Visit-2 platelet relative decline (rho=-0.313, patient-bootstrap 95% CI, -0.673 to 0.101; P=0.167, q=0.883). Box plots show the median and interquartile range, whiskers extend to the most extreme values within 1.5 interquartile ranges, and all patient values are shown. Dashed lines in the correlation panels are descriptive linear guides and are not Spearman fits. BH q values retain the complete candidate family correction. No candidate result met q <0.10; all panels are exploratory and non-mechanistic.

**Supplementary Figure S2. Paired W0-to-W10 VST transcriptomic associations with platelet change.** The analysis included 20 patients with both W0 and W10 RNA-seq; P005 was omitted only because W10 RNA was unavailable. **(A)** W0-to-W10 INFLAMMATORY RESPONSE Hallmark-score change versus Visit-3 platelet relative decline (Spearman rho=-0.362, patient-bootstrap 95% CI, -0.723 to 0.130; P=0.116, BH q=0.915). **(B)** W0-to-W10 APICAL SURFACE Hallmark-score change by Visit-3 platelet count <100 ×10⁹/L (11 vs 9 patients; event-minus-no-event median difference=-0.199, Wilcoxon P=0.0227, q=0.671). **(C)** W0-to-W10 SPP1 expression change versus Visit-3 platelet relative decline (rho=-0.311, patient-bootstrap 95% CI, -0.730 to 0.198; P=0.182, q=0.676). **(D)** Spearman correlations and patient-bootstrap 95% confidence intervals for W0-to-W10 expression changes in all six prespecified candidate genes versus Visit-3 platelet relative decline; complete values are provided in Supplementary Table S9. The box plot shows the median and interquartile range, whiskers extend to the most extreme values within 1.5 interquartile ranges, and all patient values are shown. Dashed lines in the correlation panels are descriptive linear guides and are not Spearman fits. BH q values retain the complete 50-pathway or six-gene family correction. No Hallmark or six-gene result met q <0.10; all panels are exploratory.

**Supplementary Figure S3. xCell2 cell-composition sensitivity analyses.** **(A)** Internal concordance of the primary-reference neutrophil enrichment score with matched ANC and WBC at baseline and across all visits; repeated-visit intervals use patient-cluster bootstrap. Dashed lines are descriptive least-squares guides and do not represent the Spearman fit; the displayed Spearman confidence intervals were obtained by patient bootstrap at baseline and patient-cluster bootstrap across all visits. **(B)** Paired W0-W10 neutrophil enrichment-score change in 20 patients. **(C)** Descriptive M0/M1/M2 Hallmark sensitivity overview showing, within each endpoint-model panel, the 10 pathways with the smallest raw P values; BH q values were calculated over each complete 50-pathway family without recalculation on the displayed subset. Seven baseline platelet-binary profile intervals did not converge and are flagged in the source tables. M0 includes the pathway alone; M1 adds the corresponding baseline blood count; M2 adds standardized baseline neutrophil enrichment to M1. Pathway names in panel C are abbreviated for readability; full pathway names and complete model results are provided in Supplementary Table S15. Grey cells marked with a cross indicate displayed estimates with nonconverged profile-likelihood intervals; these estimates must not be interpreted. xCell2 values are relative enrichment scores, not measured cell proportions or absolute counts.

**Supplementary Table S1. Longitudinal clinical hematology effect sizes.** For paired Visit-1-to-Visit-3 changes, the table reports median change, IQR, matched-pairs rank-biserial correlation, 10,000-resample patient-bootstrap 95% confidence intervals, and paired Wilcoxon P values. Between-group analyses report responder-minus-non-responder Hodges-Lehmann shifts and Cliff's delta with confidence intervals. Bootstrap seed was 20260920.

**Supplementary Table S2. Hematologic endpoint definitions and exact event-rate uncertainty.** Thresholds use strict less-than definitions and exact Clopper-Pearson 95% confidence intervals. The locked primary Visit-2 ANC <1.0 ×10⁹/L event count is 7/21; 6/21 is retained only as submitted-lineage sensitivity.

**Supplementary Table S3. Supportive-treatment timing and exposure-boundary audit.** The table lists deidentified timing states and the author-adjudicated primary boundary, including the three unresolved same-day visits. The post-documented oral-support-date indicator is a timing boundary, not verified continuous exposure, duration, adherence, or a causal treatment-effect estimate.

**Supplementary Table S4. Support-adjusted WBC and ANC longitudinal models.** The table reports primary and same-day boundary estimates, matched unadjusted models, denominators, exposure counts, transition counts, rank, convergence, and singularity diagnostics. Support-term estimates are non-causal because of confounding by indication and reverse causality.

**Supplementary Table S5. Strict pre-support Visit-1-to-Visit-3 sensitivity analysis.** Both scheduled CBCs were required to precede the documented support boundary. The resulting set contained five responders and four non-responders and does not replace the primary 13R/8N cohort.

**Supplementary Table S6. Baseline VST Hallmark association results.** All 50 pathways are retained within each endpoint family with effect estimates, 95% confidence intervals where estimable, raw P values, and full-family BH q values. The minimum q was 0.251; no result met q <0.10.

**Supplementary Table S7. Baseline VST candidate-gene association results.** Candidate-gene results use the DESeq2 VST primary route and retain complete family-wise BH correction. The minimum q was 0.417; results are exploratory and must not be interpreted as validated biomarkers.

**Supplementary Table S8. Paired W0-to-W10 VST Hallmark association results.** The n=20 analysis reports complete pathway families, effect estimates, 95% confidence intervals, raw P values, and BH q values. The minimum q was 0.671; no result met q <0.10. P005 is omitted only because W10 RNA is unavailable.

**Supplementary Table S9. Paired W0-to-W10 VST six-gene candidate results.** All six-gene families are retained with complete BH correction. The minimum q was 0.117. The zero median PAX6 contrast contains tied/low-information values and is not interpreted as a biomarker.

**Supplementary Table S10.** xCell2 reference and gene-overlap quality control. All 62 TPM samples were scored together. The initially drafted 0.90 shared-gene gate was changed to 0.85 before any xCell2 score was calculated or inspected; ImmuneCompendium and BlueprintEncode shared 89.64% and 87.54% of reference genes, respectively, and both passed the amended gate.

**Supplementary Table S11.** xCell2 neutrophil-score internal concordance with measured ANC and WBC. Baseline analyses contain 21 independent patients; repeated-visit intervals use a patient-cluster bootstrap over 62 observations from 21 patients. Values are relative enrichment scores, not measured cell fractions or counts.

**Supplementary Table S12.** Paired W0-W10 neutrophil enrichment-score changes. Twenty patients had paired W0 and W10 RNA-seq; P005 was omitted only because W10 RNA was unavailable. The primary-reference median change was -0.050, with a descriptive paired Wilcoxon P=0.00183.

**Supplementary Table S13.** Associations between paired neutrophil enrichment-score changes and measured ANC/WBC changes. Complete prespecified correlations for both references are reported with 2,000-resample patient-bootstrap intervals; no reference or result was selected by significance.

**Supplementary Table S14.** Baseline Hallmark M0/M1/M2 family summary. Each endpoint-model family includes all 50 Hallmark pathways. M2 adds the prespecified baseline ImmuneCompendium neutrophil enrichment score; BH adjustment is within each complete endpoint-by-model family.

**Supplementary Table S15.** Complete baseline Hallmark cell-composition sensitivity results. All 1,050 planned pathway-model rows are retained. Visit-2 platelet <100 ×10⁹/L produced 3/50 M1 and 4/50 M2 q <0.10 rows, none q <0.05; profile-interval nonconvergence and extreme interval width are retained and flagged.

**Supplementary Table S16.** Baseline Hallmark cell-composition model diagnostics. Design rank, condition number, fit convergence, and profile-likelihood interval convergence are reported for every planned model. Seven platelet-binary profile intervals did not converge and are not interpreted.

**Supplementary Table S17.** Paired W0-W10 Hallmark composition-change sensitivity family summary. The reviewer-directed extension contains 20 paired patients, three hematologic lineages, three model variants, and all 50 pathways per family. All 450 models were identifiable and converged; no family contained a q <0.10 pathway.

**Supplementary Table S18.** Complete paired W0-W10 Hallmark composition-change sensitivity results. M2 adds W10-minus-W0 primary-reference neutrophil enrichment-score change. All planned rows and diagnostics are retained without significance-based filtering; these concurrent associations are not causal correction or mediation estimates.

**Supplementary Table S19. Clinical sensitivity analysis excluding the sole baseline HBeAg-positive participant.** The all-patient primary analysis (21 participants: 13 responders and 8 non-responders) is presented alongside the original exclusion sensitivity (20 participants: 13 responders and 7 non-responders). P021 remains in the primary cohort and no participant was reclassified. Within-group summaries describe Visit 1 and Visit 3 values and the median within-person change (Visit 3 minus Visit 1); between-group summaries describe observed nadirs and relative declines. Observed nadirs use all eligible records in the locked clinical dataset; relative decline is (baseline − nadir)/baseline and is reported as a proportion. WBC, ANC, and PLT counts are in ×10⁹/L. P values are from two-sided Wilcoxon signed-rank tests for paired comparisons and rank-sum tests for between-group comparisons, using asymptotic inference with continuity correction. Nominal P values are unadjusted and descriptive; no equivalence, causal effect, or external validity is established. There were no missing required measurements in these comparisons.
